# To Tube or Not to Tube? The Role of Intubation during Stroke Thrombectomy

**DOI:** 10.3389/fneur.2014.00170

**Published:** 2014-09-23

**Authors:** Courtney Takahashi, Conrad W. Liang, David S. Liebeskind, Jason D. Hinman

**Affiliations:** ^1^Department of Neurology and Neurocritical Care, Oregon Health and Science University, Portland, OR, USA; ^2^Department of Neurology, David Geffen School of Medicine, University of California Los Angeles, Los Angeles, CA, USA

**Keywords:** acute stroke, thrombectomy, intubation, endovascular therapy, clot retrieval

## Abstract

In the 10 years since the FDA first cleared the use of endovascular devices for the treatment of acute stroke, definitive evidence that such therapy improves outcomes remains lacking. The decision to intubate patients undergoing stroke thrombectomy impacts multiple variables that may influence outcomes after stroke. Three main areas where intubation may deleteriously affect acute stroke management include the introduction of delays in revascularization, fluctuations in peri-procedural blood pressure, and hypocapnia, resulting in cerebral vasoconstriction. In this mini-review, we discuss the evidence supporting these limitations of intubation during stroke thrombectomy and encourage neurohospitalists, neurocritical care specialists, and neurointerventionalists to carefully consider the decision to intubate during thrombectomy and provide strategies to avoid potential complications associated with its use in acute stroke.

## Introduction – Thrombectomy and Intubation

In 2004, the FDA cleared the use of the first clot retrieval device, the MERCI retriever, for thrombectomy in acute ischemic stroke up to 8 h after symptom onset (1). A variety of modalities have been adapted for intracranial thrombectomy including corkscrew retrieval devices, aspiration thrombectomy, and stent retrievers. These devices are able to mechanically remove clots that obstruct blood flow in the intracranial vasculature (2). While a relatively small percentage of all stroke patients undergo mechanical thrombectomy, this number has increased sixfold from 0.1% of all stroke cases in 2004 to 0.6% of all stroke cases in 2009 (3).

Despite the initial excitement regarding the possibilities afforded by mechanical thrombectomy, the results of recent large randomized trials have been disappointing. Three large trials have failed to demonstrate the superiority of thrombectomy over tPA that had been previously expected (4–6). One reason for the unexpected findings may be related to the high rates of intubation that occurred when thrombectomy was introduced. Routine intubation has traditionally been preferred by interventionalists because it reduces pain and discomfort for the patient and prevents movement during intracranial catheter navigation and clot retrieval. This improves the ease of performing the procedure and minimizes the potential risk for iatrogenic vessel dissection or perforation. When thrombectomy first became available, most cases were performed with general anesthesia; very few patients received conscious sedation. A survey of interventionalists showed a preference for performing most cases under general anesthesia (7). These early studies, in which intubated patients were overly represented may not have captured all the complications associated with intubation. Most recently, the IMS3 trial data, which were collected prospectively, but retrospectively analyzed, showed the increased risks associated with intubation. Among 434 patients, 269 (62%) had their procedure performed under local anesthesia, while 147 (33.9%) received general anesthesia. Except for slightly milder strokes in the local anesthesia group (NIHSS 16 vs. 18), the groups were evenly matched.

Several years after the introduction of thrombectomy, centers began to utilize conscious sedation, rather than rely exclusively on general anesthesia. Hence, more recent studies comparing outcomes in intubation likely had more balanced groups, where the number of intubated and non-intubated patients was more equal. When conscious sedation was compared to general anesthesia, investigators ultimately discovered that intubation was associated with higher rates of sub-arachnoid hemorrhage and increased mortality (8). Large, retrospective studies have linked intubation to worsened outcomes after thrombectomy, including longer hospital stays and poorer functional outcomes in two large studies (9, 10).

**Figure 1:**
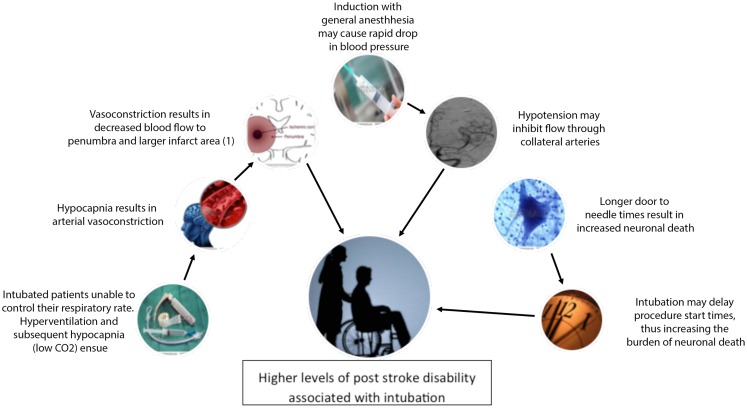
**Diagram of proposed relationships between general anesthesia and observed higher levels of post stroke disability**. (1) Reprinted by permission from Macmillan Publishers Ltd: Acta Pharmacologica Sinica, Reference 3460241439635 copyright 2011.

Intubating patients during thrombectomy may actually be worsening outcomes by changing cerebral blood flow patterns, increasing minute ventilation, or delaying revascularization (11, 12) (Figure 1). In this mini-review, we review the current data on these potential complications of intubation and make a case for avoiding intubation whenever possible during acute stroke thrombectomy.

## Time is Brain

One robust finding, across multiple clinical stroke trials and animal models, is the notion that “time is brain.” In essence, shortly after a clot blocks an artery, ischemia ensues very quickly and the stroke area rapidly grows. While the “time is brain” approach assumes a linear relationship between time and brain ischemia where there likely is not one, an abundance of basic science and clinical data supports the effort to emphasize rapid treatment of stroke. A pooled analysis of several large stroke trials showed a significant benefit (*p* < 0.02) the earlier tPA is administered, with patients receiving tPA in <90 min conferring the highest benefit (13). The earlier IMS trials demonstrated a 10% decrease in the likelihood of good outcomes for every 30 min of case delay (14). A pooled analysis of six major stroke trials also showed that the number needed to treat (NNT) was significantly lower the closer patients were to their symptom onset (15) (Table 1).

**Table 1:** **Relationship between time of symptom onset and functional. outcome (measured by modified Rankin scale), after reperfusion therapy with IV-tPA**.

Time (minutes after onset)	Number needed to treat	Number needed to harm
0–90	3.6	65
91–180	4.3	38
181–270	5.9	30
271–360	19.3	14

*Table based on work by Lansberg et al. (15)*.

While time to treatment was not a primary end point in IMS3, the study still demonstrated the utility of early revascularization. Patients who received tPA in <90 min had better outcomes and patients who received earlier thrombectomy had a trend toward better outcomes (16). Another study, produced using the IMS3 data, demonstrated that in 240 proximal vessel occlusions, angiographic reperfusion (defined as TICI score 2–3) was achieved in 182 (76%) of all patients. The average time to reperfusion was 325 min; the range varied from 180 to 418 min. Increased times to reperfusion were associated with worse clinical outcomes – every 30 min delay decreased the likelihood of good clinical outcome (16). Data from the STAR study also show that every 60 min increase in onset to reperfusion time decreased good outcome by 38% (17). Similarly, the RECANALISE trial, a prospective study comparing outcomes in patients with confirmed arterial occlusions treated with IV-tPA vs. IV-tPA plus endovascular therapy, concluded that door to groin puncture times influenced outcomes. In the IV-tPA plus thrombectomy arm, patients who received their intervention in <210 min were more likely to have a better outcome (defined as mRS <2 at 3 months, NIHSS 0–1 24 h post stroke, or reduction of four NIHSS points 24 h after admission) compared to patients who received treatment after 210 min. Every 30 min reduction in onset to reperfusion times was associated with increased likelihood of good clinical outcome in RECANALISE (18). A pooled analysis from seven different centers demonstrated a significant association and trend toward increased mortality as onset to reperfusion times increased (*p* < 0.001, adjusted OR 1.19, 95% CI 1.05–1.35). Intracerebral hemorrhage was associated with longer onset to reperfusion time. Not surprisingly, favorable and excellent outcomes were associated with shorter onset to reperfusion times. Overall, this pooled analysis concluded a 20% decreased likelihood of good outcome with every 30 min delay (15).

One major disadvantage to intubation prior to thrombectomy is the delay in procedure start times. In practical terms, intubation increases the door to groin puncture times. Given the rich data that support better outcomes with shorter symptom onset to treatment times, unnecessary intubations may result in delayed treatment times and ultimately higher post stroke disability. A recent study in Germany examined both retrospective and prospective door to groin puncture times, after a fast-track intubation system had been implemented. Prior to the system implementation, intubation delayed procedure onset times by an average of 51 min. After the implementation of fast-track program, door to groin puncture times were improved (25 ± 10 min) but still slower than the times of patients who arrived previously intubated (19). Hence, even when streamlined programs in tertiary care centers are implemented, intubation may produce an unnecessary delay in procedural start times. At present, the best approach would be to recommend intubation in patients who may suffer from any type of cardiorespiratory compromise or suspected impending airway failure. A streamlined system for intubation is ideal, as it would be the best way to reduce door to needle times. Hospitals should strive to reduce intubations for the purpose of procedural ease, as the current evidence suggests that these procedures may delay thrombectomy start times and ultimately result in patient harm (20).

## Effects of Intubation on Systemic Blood Pressure

The optimal blood pressure goals in the acute post stroke period are not clearly defined and remain a controversial topic among neurologists (21). *Post hoc* analysis of the International Stroke Trial reported a “*U*”-shaped curve when relating initial blood pressures to outcome – patients with very low and very high blood pressures tended to have the worst outcomes (22). While it may be beneficial in some cases to treat elevated blood pressures in the acute post stroke period (23, 24), it is *not* beneficial to lower blood pressures so that the patient becomes systemically hypotensive. The initial cerebral response to an arterial blockage is an increase in systemic blood pressure. This increase is transient as the brain seeks to shunt blood around the blockage by augmenting collateral flow through smaller arteries and arterioles to perfuse the affected stroke territory (25).

Many general anesthetic agents lower blood pressure and even cause frank hypotension (25). Most of the induction agents, including propofol, one of the most commonly utilized induction agents, include hypotension as a side effect. While ketamine and etomidate are the best agents to maintain blood pressure, their use may be limited. Ketamine can cause post operative psychosis and etomidate is associated with increased 30 day mortality and cardiac morbidity (26, 27). Therefore, lower systemic blood pressures and resultant lower cerebral perfusion pressures caused by vasodilating general anesthetics may be deleterious to outcomes (25).

In practice, however, the effect of blood pressure in the acute stroke period and the effects of peri-procedural blood pressures are difficult to determine. Data on the control of blood pressure in stroke patients treated with endovascular thrombectomy are limited and reveal mixed results. Some small, retrospective studies have not shown effects between blood pressure and outcomes. For instance, a pooled analysis performed on patients treated with the MERCI device showed that higher pre-treatment systolic blood pressures were associated with lower revascularization rates. The study dichotomized blood pressures into groups >150 vs. <150 mmHg systolic and found that higher initial blood pressures resulted in significantly worse rates of revascularization (28). Similarly, a single retrospective study of acute stroke patients treated with mechanical thrombectomy looked at the relationship between changes in blood pressure and functional outcome. The study patients were acute stroke patients who received mechanical thrombectomy for treatment of their stroke. Outcomes were dichotomized into “good” outcomes (mRS < 4) vs. “poor” outcomes (mRS ≥ 4). While there was a significant drop in blood pressure in both groups after induction with general anesthesia, there was no association between blood pressure drops and functional outcomes at 3 months (11).

However, in a similar single center, retrospective Canadian study investigators reported an association between low systolic blood pressures (SBP < 140) and worsened functional outcomes. Functional outcomes were measured by modified Rankin score, with score 0–2 considered a “good” outcome. The study demonstrated lower blood pressures, particularly the lowest blood pressure readings, in intubated patients who received general anesthesia. The study also attempted to control for other factors that may affect outcome after stroke through the use of binary logistic regression analyses to adjust for other factors known to affect stroke outcomes (age, diabetes, and heart disease), and even considered highest and lower systolic and diastolic blood pressures. Using this rigorous approach, the study found an association between lower intraprocedural blood pressures and outcome (29).

Concrete recommendations regarding blood pressure management during and after thrombectomy do not exist. Current studies are small and retrospective in nature, yet, it is likely that hypotension and blood pressure lowering in normotensive patients appears harmful, particularly, in view of the AHA/ASA guidelines that recommend anti-hypertensive treatment only for patients with sustained systolic blood pressures over 220 mmHg in the setting of acute ischemic stroke (30). While consensus data on patients undergoing thrombectomy does not exist, there is limited clinical data and pathophysiologic mechanisms to suggest that anesthetic-induced hypotension in peri-procedural period likely blunts the presumed protective response in acute stroke.

## Strategies to Minimize Blood Pressure Drops

Reperfusion therapies such as thrombectomy are designed to improve cerebral blood flow, but intubation and adjunctive medications used for intubation frequently worsen cerebral hemodynamics. Since blood pressure drops are inevitable with induction, minimizing the use of general anesthesia may be one way to limit blood pressure changes. Avoidance of the use of the halogenated anesthetic agents and propofol may result in better outcomes, since both classes of drugs can cause hypotension through systemic vasodilation (31, 32). An alternative may be etomidate, as it is less associated with dramatic blood pressure drops (33). Aggressive treatment of dehydration or under-resuscitation and hypotension associated with infection may also help to improve patient outcomes, though this has not been rigorously evaluated with trial data (28, 30). Hence, when intubation is necessary, we would recommend setting strict blood pressure guidelines for the anesthesiologist at the beginning of thrombectomy and augmenting anesthetic induction with fluid or pressor support as needed to maintain a MAP no <15% of the pre-procedural MAP.

## Hypocapnia and Its Effects on Cerebral Blood Flow

Intubation and general anesthesia may also result in worsened neurological outcomes in acute stroke patients because of the effect of hypocapnia on the arterial system of the brain. Intubated patients are unable to control their respiratory rate – their respiratory rates are instead controlled by ventilator parameters and inversely related to arterial CO_2_ levels (12, 31, 34). By surrendering patients’ CO_2_ levels to a ventilator, the patient may become relatively hypocapnic, suffer from vasoconstriction, and ultimately a larger stroke territory with resultant worse functional outcomes (35). Studies of both EMS providers and anesthesiologists show that providers have a tendency to hyperventilate neurologically injured patients (36, 37). Hyperventilation may ultimately lead to hypocapnia, which causes arterial vasoconstriction and this may cause decreased perfusion to the ischemic penumbra (12, 34). Even small differences may have consequences – a 1 mmHg decrease in CO_2_ may result in a 3% decrease in arterial flow (34).

Animal models demonstrate that hypocapnic states produce dysregulation and decreased cerebral blood flow as well. Newborn piglet studies show that hypercapnic vasodilation is lost after ischemia reperfusion. Interestingly, vasoconstriction in response to hypocapnia is maintained (38). Rat models have also shown caudoputamenal damage in response to hypocapnic states associated with mechanical ventilation (39). Human patients affected by stroke also show widespread vasoconstriction, even in the hemisphere contralateral to the stroke territory (40–42).

Clinical evidence also implies that hypocapnia may be associated with worse clinical outcomes. In *post hoc* analysis, a small single center retrospective study found an association between lower CO_2_ levels and worsened post stroke functional outcomes (11). Hypocapnia may cause problems with blood vessel reactivity throughout the cerebral circulation. Small studies of stroke patients have demonstrated abnormal vessel reactivity in the hemisphere contralateral to the stroke, implying that hypocapnia may cause widespread depressed cerebral blood flow (43). Another investigation suggested that in the acute stroke setting, arteries may be able to vasoconstrict in response to hypocapnia but they are unable to vasodilate in response to elevated CO_2_ levels (44). Anesthesiologists may decide to manage a case with low normal CO_2_ levels or mild hypocapnia because they fear normocapnia and hypercapnia will exacerbate intracranial pressures. Anesthesiologists may also favor keeping patients slightly hypocapnic because they often rely on end tidal monitors to evaluate ventilation during the case. Because end tidal monitors are unable to account for CO_2_ trapped in dead spaces of the lung, end tidal values are typically lower than arterial CO_2_. The difference between end tidal and arterial CO_2_ levels is even wider when patients have pre-existing cardiopulmonary disease. Hence, when relying on end tidal monitors, anesthesiologists may target lower CO_2_ values, presuming that the arterial values are higher (45, 46).

While there is no definitive evidence such as a large, randomized control trial regarding the effects of hypocapnia in acute stroke, animal evidence and small studies suggest that it may have deleterious consequences. Hypocapnia may cause vasoconstriction, while hypercapnia can increase intracranial pressures; hence, until a randomized human trial is completed, normocapnia is likely the safest ventilation strategy (12, 26).

## Conclusion

In the acute stroke setting, recent retrospective evidence suggests that patients who receive conscious sedation fare better than those who receive general anesthesia and intubation. This observation may explain, at least in part, the worsened outcomes experienced by thrombectomy patients when compared to tPA alone (4, 47). The underlying mechanism may be related to delayed treatment times, drops in systemic blood pressure that compromise the ischemic penumbra or hypocapnia with its associated arterial vasoconstriction. More recent studies posit that the worsened outcomes may be related to higher risk for aspiration pneumonia and risk of sepsis in intubated vs. non-intubated patients (48). Regardless of mechanism, the present studies, albeit most of them retrospective, suggest increased mortality associated with intubation (9, 10, 49). Likely these contributing factors synergize to negatively impact patient outcome and should be considered in future clinical trials of acute stroke management using thrombectomy. The decision to intubate an acute stroke patient should not be enacted automatically or by institutional ischemic stroke algorithm. Rather this decision should be considered carefully by the stroke neurologist, neurointerventionalist, and neurocritical care physicians on an individual patient basis.

## Conflict of Interest Statement

The authors declare that the research was conducted in the absence of any commercial or financial relationships that could be construed as a potential conflict of interest.
